# Design of a Type-1 Diabetes Vaccine Candidate Using Edible Plants Expressing a Major Autoantigen

**DOI:** 10.3389/fpls.2018.00572

**Published:** 2018-05-01

**Authors:** Edoardo Bertini, Matilde Merlin, Elisa Gecchele, Andrea Puggia, Annalisa Brozzetti, Mauro Commisso, Alberto Falorni, Vittorio Bini, Victor Klymyuk, Mario Pezzotti, Linda Avesani

**Affiliations:** ^1^Department of Biotechnology, University of Verona, Verona, Italy; ^2^Department of Medicine, University of Perugia, Perugia, Italy; ^3^Icon Genetics GmbH, Halle, Germany

**Keywords:** oral tolerance, autoimmune diabetes, immunotherapy, transient expression, red beet, molecular farming, GAD65, magnICON^®^

## Abstract

Type-1 diabetes (T1D) is a metabolic disease involving the autoimmune destruction of insulin-producing pancreatic beta cells. It is often diagnosed by the detection of autoantibodies, typically those recognizing insulin itself or the 65-kDa isoform of glutamic acid decarboxylase (GAD65). Oral insulin can be used to induce systemic immunological tolerance and thus prevent or delay the onset of T1D, suggesting that combination treatments with other autoantigens such as GAD65 could be even more successful. GAD65 has induced oral tolerance and prevented T1D in preclinical studies but it is difficult to produce in sufficient quantities for clinical testing. Here we combined edible plant systems, namely spinach (*Spinacia oleracea* cv Industra) and red beet (*Beta vulgaris* cv Moulin Rouge), with the magnICON^®^ expression system to develop a safe, cost-effective and environmentally sustainable platform for the large-scale production of GAD65. The superior red beet platform was extensively characterized in terms of recombinant protein yields and bioequivalence to wild-type plants, and the product was tested for its ability to resist simulated gastric digestion. Our results indicate that red beet plants are suitable for the production of a candidate oral vaccine based on GAD65 for the future preclinical and clinical testing of T1D immunotherapy approaches.

## Introduction

Type 1 diabetes (T1D) is a disorder of glucose metabolism caused by the autoimmune destruction of insulin-producing pancreatic beta cells. Currently, the only effective way to manage the disease is lifelong daily insulin injections. There are ∼422 million diabetics worldwide, with T1D accounting for 5–10% of this population (You and Henneberg, 2016). Diabetic complications are a major cause of morbidity and mortality in T1D patients, and alternative treatments that prevent or delay the onset of T1D are therefore highly desirable.

Several immunological approaches have been explored to reduce the burden of T1D, and the induction of systemic immunological tolerance by the oral administration of autoantigens is the most promising strategy due to the specificity of the treatment and the negligible side effects. T1D is often diagnosed by the detection of autoantibodies against insulin itself (specifically preproinsulin), the 65-kDa isoform of glutamic acid decarboxylase (GAD65), the phosphatase IA-2 and/or the zinc transporter ZnT8 (Mauvais et al., 2016). These principal autoantigens could be used for the induction of oral tolerance but massive quantities of each protein are needed, and the treatment is only effective in narrow dose ranges (Mowat et al., 2004).

The production of autoantigens for the induction of oral tolerance requires a safe, highly scalable and cost-effective heterologous production system. Plants meet these criteria because they do not support the replication of mammalian pathogens, production can be scaled up easily, and plants do not require the expensive infrastructure associated with cell-based systems, i.e., stainless-steel fermenters (Tusé et al., 2014). Furthermore, edible plant tissues engineered to produce recombinant proteins can be used for the direct oral administration of autoantigens without extensive purification, which is beneficial because up to 80% of biopharmaceutical manufacturing costs relate to downstream processing (Wilken and Nikolov, 2012; Buyel et al., 2015). The ability to grow antigen-producing plants locally also eliminates the need for transportation and a cold chain (Kwon et al., 2013). Among the various strategies developed for the expression of recombinant proteins in plants, transient expression in leafy crops using deconstructed plant viruses achieves a superior performance in terms of yields and speed, allowing the production of large quantities of target proteins within a short time (Rybicki, 2010). In particular, the magnICON^®^ system has been extensively used in *Nicotiana benthamiana* as the gold standard production host, but this tobacco-related species is not an ideal basis for oral vaccines because alkaloids and other metabolites accumulate in the leaves (Merlin et al., 2016).

GAD65 is currently under investigation in human clinical trials as a means to prevent or delay T1D by inducing oral tolerance (Ludvigsson, 2013). GAD65 has already been expressed in several plant systems, and the catalytically inactive version of the protein (GAD65mut) was found to be more stable than the wild-type form, resulting in 10-fold higher yields (Avesani et al., 2010). Furthermore, we have previously shown that a truncated form of GAD65mut lacking the first 87 amino acids (Δ87GAD65mut) is more soluble than GAD65mut and accumulates to higher levels in leaves when expressed using the magnICON^®^ system (Merlin et al., 2016). Here we combined the superior performance of the magnICON^®^ system with two edible plant species to develop a new platform for the large-scale production of GAD65 in plant tissue, in order to determine the feasibility of edible plants as a means to induce oral tolerance against T1D in preclinical and clinical studies.

## Materials and Methods

### Construction of Plant Expression Vectors

The magnICON^®^ Tobacco mosaic virus 3′ module pICH31070, containing the GAD65mut and Δ87GAD65mut sequences, was prepared as previously described (Avesani et al., 2014; Merlin et al., 2016). The final vectors pICH31070.Δ87GAD65mut, pICH31070.GAD65mut, and pICH7410.GFP (3′ modules), pICH20111 (5′ module) and pICH14011 (integrase module) were introduced into *Agrobacterium tumefaciens* strain GV3101 by electroporation.

### Transient Expression in Edible Plants

Spinach and red beet plants were grown in a growth chamber (day/night temperatures of 23/21°C, 12-h photoperiod, 65% humidity). Five-week-old spinach and six-week-old red beet plants were used for both syringe and vacuum agroinfiltration. The bacteria were seeded into lysogeny broth (LB) medium containing 50 μg/mL rifampicin and 50 μg/mL kanamycin (pICH31070.Δ87GAD65mut and pICH31070.GAD65mut) or 50 μg/mL carbenicillin (pICH14011, pICH20111 and pICH7410.GFP). For syringe agroinfiltration, overnight bacterial cultures were collected by centrifugation at 4500 × *g* and resuspended in two volumes of infiltration buffer (10 mM MES pH 5.5, 10 mM MgSO_4_). Following incubation for 3 h at room temperature, the GAD65mut, Δ87GAD65mut or GFP 3′ module suspension was mixed with equal volumes of the 5′ module and integrase module suspensions and the mixture was used to infiltrate the leaves of spinach and red beet plants, with each biological replicate comprising a pool of three infiltrated leaves from different plants, sampled from 4 to 12 dpi for GFP and from 2 to 14 dpi for the GAD65 forms. A mixture of the 5′ module and integrase module suspensions was used as a negative control. For vacuum agroinfiltration, the bacteria were inoculated in 200 mL of selective LB medium and grown to saturation. The overnight culture was pelleted at 4500 × *g* for 20 min and resuspended in infiltration buffer to an OD_600_ of 0.35, 0.035 or 0.0035 (corresponding to 10^-1^, 10^-2^, and 10^-3^ dilutions, respectively). Following incubation for 3 h at room temperature, the GAD65mut, Δ87GAD65mut or GFP 3′ module suspensions were mixed with equal volumes of the 5′ module and integrase module suspensions and vacuum infiltrated into the aerial parts of red beet plants dipped into the infiltration suspension in a vacuum chamber (Thermo Fisher Scientific, Waltham, MA, United States). Vacuum was applied for 3–5 min using a VCP 80 pump (VWR, Radnor, PA, United States) with a pressure of 90 mBar. Vacuum release was maintained for 45 s. Each biological replicate comprised the infiltrated leaves from a single plant, sampled at the maximum expression peak for each recombinant protein. In some experiments, the detergent Tween-20 (Sigma-Aldrich, St Louis, MO, United States) was added to the infiltration suspension at different concentrations (0.005, 0.01, and 0.05%). After both agroinfiltration procedures, plants were returned in a growth chamber under standard conditions.

### Visualization and Absolute Quantification of GFP

Leaves expressing GFP were viewed under UV illumination using a B-100AP lamp and GFP was quantified using a VICTOR^3^ Multilabel Counter (model 1420-011, PerkinElmer, Waltham, MA, USA). The absolute GFP protein concentration was determined by comparing different dilutions of total soluble protein extracts to a standard curve prepared using recombinant GFP (Roche Applied Science, Penzberg, Germany).

### Analysis of Recombinant Protein Expression

Total soluble proteins were extracted from leaves by grinding tissue samples to fine powder under liquid nitrogen. The powder was resuspended in three volumes of extraction buffer (50 mM sodium phosphate pH 8.0, 20 mM sodium metabisulfite, 0.5% Tween-20) supplemented with Protease Inhibitor Cocktail (Sigma-Aldrich). The same procedure was used for freeze-dried and dried leaves, excluding the grinding under liquid nitrogen. The homogenate was centrifuged at 30,000 × *g* for 40 min at 4°C. Protein levels were determined using the Quantum Protein Bicinchoninic Protein Assay Kit (EuroClone, Pero, Italy). For western blot analysis, equal volumes of total soluble protein were boiled for 10 min, then separated by SDS-PAGE using 10% polyacrylamide for GAD65 variants and 12% polyacrylamide for GFP, and transferred to a nitrocellulose membrane by electroblotting. The GAD65 variants were detected using an anti-GAD65/67 antibody (G5163, Sigma-Aldrich) diluted 1:10 000, which recognizes the C-terminal linear peptide shared by GAD65 and GAD67, GFP was detected using the anti-GFP antibody ab290 (Abcam, Cambridge, United Kingdom) diluted 1:20 000, and LHCB2 was detected using a specific antibody kindly provided by Prof. R. Bassi, diluted 1:10,000, all followed by incubation with a secondary horseradish peroxidase-conjugated rabbit anti-mouse IgG, diluted 1:10,000 (A6154, Sigma-Aldrich). The signal was detected using ECL Select western blotting detection reagent (GE Healthcare, Little Chalfont, United Kingdom). The ChemiDoc^TM^ system (Bio-Rad Laboratories, Hercules, CA, United States) was used to capture chemiluminescent signals and images were analyzed using Quantity One 1-D Analysis Software (Bio-Rad). The relative and absolute levels of the GAD65 variants were determined using different concentrations of recombinant human GAD65 (Dyamid Diagnostics, Stockholm, Sweden).

### Radiobinding Assay for GAD65 and Δ87GAD65mut Autoantibodies

The immunoreactivity of Δ87GAD65mut was evaluated by testing autoantibodies against wild-type GAD65 and Δ87GAD65mut in serum samples from 94 subjects newly diagnosed with T1D (recruited consecutively between 2006 and 2016) and from 106 healthy control subjects.

The Δ87GAD65mut sequence was cloned in the pCR^TM^ II TOPO vector using the TOPO TA cloning kit (Thermo Fisher Scientific) according the manufacturer’s instructions. The fragment was initially amplified from pENTR.Δ87GAD65mut as a template using Platinum high-fidelity Taq DNA polymerase (Thermo Fisher Scientific), forward primer- 5′-AAG AAT TCA ATT CAC CAT GAA CTA CGC GTT TCT CCA T-3′ and reverse primer 5′-CGT CTA GAT TAT TAT AAA TCT TGT CCA AGG CGT-3′. The resulting plasmid was introduced into chemically competent One-shot TOP 10 *Escherichia coli* cells (Thermo Fisher Scientific). Transformants were checked for the correct insert using the cloning primers and M13 primers provided in the TOPO TA kit, and another primer pair identifying a 147-bp fragment within the Δ87GAD65mut clone (forward primer 5′-AGG GAT TGA TGC AGA ATT GC-3′ and reverse primer 5′-CCC TCC ACA TCA GCC ATA GT-3′). Two colonies among those positive for the correct insert were grown overnight in LB medium containing 50 μg/mL kanamycin and plasmid DNA was purified using a commercial kit (Qiagen, Hilden, Germany).

GAD65 and Δ87GAD65mut were ^35^S-radiolabeled by coupled *in vitro* transcription and translation using rabbit reticulocyte lysate (Promega Corp., Madison, WI, United States) and ^35^S-methionine (PerkinElmer) according to the manufacturer’s instructions. In the autoantibody assays, 20,000 cpm of ^35^S-GAD65 or ^35^S-Δ87GAD65mut was immunoprecipitated in duplicate with human serum at a dilution of 1:25. The immunocomplexes were separated in 96-multiwell plates (Millipore, Bedford, MA, United States) using Protein A-Sepharose (GE Life Sciences, Uppsala, Sweden) as previously described (Falorni et al., 1995). Finally, immunoprecipitated radioactivity was evaluated in a Top Count-NXT microplate scintillation counter (Packard Bioscience Co., Meriden, CT, United States) against a positive standard serum and two negative standard sera. Levels of autoantibodies were expressed as a relative index according to the following formula: (cpm sample – mean cpm negative controls)/(cpm positive controls – mean cpm negative controls). The area under a ROC curve for diagnostic sensitivity and specificity was determined and the best cut-off value for each assay was calculated according to the Youden index (Cantor and Kattan, 2000).

### Plant Material Processing

Vacuum agroinfiltrated red beet leaves expressing Δ87GAD65mut sampled at the expression peak were frozen in liquid nitrogen, stored at -80°C, lyophilized for 72 h using an LIO5P 4k freeze-dry system in a vacuum at -50°C and 0.036 mBar, or dried in a stove at 50°C for 10–11 h. Lyophilized and heat-dried leaf material was ground into fine powder using respectively an ordinary coffee grinder or by mortar and pestle. The powder was stored in a sealed container with silica gel at room temperature to exclude moisture.

### Simulation of Gastric Digestion

Gastric digestion was simulated by vortex homogenizing 100 mg of grinded freeze-dried red beet leaves expressing Δ87GAD65mut, obtained as described in the previous paragraph, in 6 mL PBS (8 g/L NaCl, 0.2 g/L KCl, 1.44 g/L Na_2_HPO_4_, 0.24 g/L KH_2_PO_4_ pH 7.4). The samples were adjusted to pH 2 with 6 M HCl and 4 mg/mL pepsin from porcine gastric mucosa (P7012, Sigma-Aldrich) in 10 mM HCl to obtain a final pepsin concentration of 1 mg/mL or a ratio of 1:20 to total soluble protein. The samples were shaken for 120 min at 37°C, adjusted to pH 6 with 1 M NaOH to inactivate pepsin. 750-μL aliquots were centrifuged at 20,000 × *g* for 30 min at 4°C and the supernatant and pellet samples were both analyzed by western blot analysis as described above. To analyze the pellet, it was resuspended in one supernatant volume of loading buffer. A sample treated according to the protocol above but without pepsin was used as a negative control. Three biological replicates were prepared for every sample. For the analysis of cell integrity, the freeze-dried and heat-dried samples were processed as above, but without the pepsin solution and at different pH values (7.4 or 2). This analysis was performed using one biological replicate for each sample.

### Bioburden Assessment

We homogenized 100 mg of freeze-dried red beet leaves expressing Δ87GAD65mut (or non-infiltrated controls) in 8 ml sterile PBS (pH 7.4) vortexed for 1 min and transferred 1 ml onto LB medium without antibiotics or containing (i) 50 μg/mL rifampicin, (ii) 50 μg/mL each of rifampicin and carbenicillin, (iii) 50 μg/mL each of rifampicin and kanamycin, or (iv) 50 μg/mL each of rifampicin, carbenicillin and kanamycin. The residual bacterial charge was examined after 3 days at 28°C and represented as colony forming units (CFU)/mL. Three biological replicates were tested for each sample.

### Extraction of Metabolites

For the extraction of primary metabolites, infiltrated and non-infiltrated red beet plants were processed as described by Antonio et al. (2007) with modifications. Leaves were ground to powder in liquid nitrogen and 30 mg of the leaf powder was extracted by vortexing for 30 s with 750 μl ice-cold 7/3 (v/v) methanol/chloroform. After incubation at -20°C for 2 h, we added 600 μl of cold water and the extracts were centrifuged at 17900 × *g* at 4°C for 10 min. The upper hydroalcoholic phase was collected and evaporated in a Heto Holton Maxi-Dry Plus Vacuum (Thermo Fisher Scientific). The pellets were resuspended in 300 μl of 50/50 (v/v) acetonitrile/water as previously described (Antonio et al., 2008). Samples were sonicated for 3 min and passed through 0.2-μm Minisart RC4 membrane filters (Sartorius-Stedim, Göttingen, Germany). For the extraction of secondary metabolites, 300 mg of the leaf powder was processed essentially as described by Commisso et al. (2017) but using 100% methanol as the solvent. Polar lipids were extracted from 200 mg of leaf powder as described by Negri et al. (2017). Prior the final filtration, supernatants were diluted 1:5 with methanol, sonicated for 3 min and centrifuged at 17900 × *g* at 4°C for 10 min. The supernatants were passed through 0.2-μm Minisart RC4 filters as above.

### LC-MS Analysis

Samples were analyzed at 0.2 mL/min by high-performance liquid chromatography (HPLC) using a Beckman Coulter 508 autosampler (Beckman Coulter, Fullerton, CA, United States) maintained at 4°C, coupled with a Beckman Coulter Gold 127 HPLC device. Primary metabolites (5 μL injection volume) were separated using a VisionHT HILIC guard column (7.5 × 2.1 mm, 3 μm particle size; Grace, Columbia, MD, USA) in line with an Ascentis Express HILIC column (150 × 2.1 mm, 2.7 μm particle size; Supelco, Sigma-Aldrich). The solvents were water plus 20 mM ammonium formiate (A) and 95% acetonitrile, 5% water plus 10 mM ammonium formiate (B). Starting at 100% B for 10 min, the gradient declined to 85% B in 15 min followed by a hold for 5 min, then to 50% B in 10 min followed by a hold for 30 min, and then increased to 100% B in 1 min with a final equilibrium for 29 min. Secondary metabolites and polar lipids (10 μL injection volume) were analyzed using a C18 guard column (7.5 × 2.1 mm, 5 μm particle size) in line with an HP C18 column (150 × 2.1 mm, 3 μm particle size) both sourced from Alltima (Nicholasville, KT, United States). The solvents were water plus 0.05% formic acid (A) and acetonitrile plus 0.05% formic acid (B) for secondary metabolites, or 100% acetonitrile (B) for polar lipids. Samples were separated following gradients as reported by Commisso et al. (2017) for secondary metabolites and by Negri et al. (2017) for polar lipids.

The HPLC system was coupled to an Esquire 6000 ion trap mass spectrometer (Bruker Corp., Billerica, MA, United States) equipped with an electrospray ionization (ESI) source for the analysis of primary and secondary metabolites, whereas atmospheric-pressure chemical ionization (APCI) was used to detect polar lipids. Samples were ionized in negative and positive modes. The ESI values were 50 psi and 350°C for the nitrogen nebulizing gas and 10 L/min for the drying gas. For APCI, the same values were used and the vaporizer was set at 450°C. The mass spectrometer scanned the 50–1500 *m/z* range and the target masses were set at 200 *m/z* for primary metabolites, 400 *m/z* for secondary metabolites and 700 *m/z* for polar lipids. LC-MS data were recorded and processed with Esquire Control v5.2 and Data Analysis v3.2 (Bruker Corp.), respectively. The latter was used to convert.d raw data into net.cdf files. MZmine v2^1^ was used for the detection, deconvolution and alignment of the *m/z* features to create the data matrices used for multivariate statistical analysis. Quality controls were prepared by mixing equal parts of wild-type, negative control and Δ87GAD65mut extracts. These samples were analyzed at the beginning, in the middle and at the end of each experiment.

### Statistical Analysis

For the autoantibody assays, diagnostic sensitivity was calculated as the percentage of T1DM sera that scored positive and diagnostic specificity as the percentage of healthy control sera that scored negative. Diagnostic accuracy was calculated as the AUC for a binary diagnostic test (positive/negative) (Cantor and Kattan, 2000). The concordance of qualitative results between different determinations was determined using the Cohen’s κ coefficient of inter-rater agreement according to the classification of sera as positive or negative (Altman, 1995). Cohen’s κ coefficient provides a measure of the overlapping of classifications by different methods, and the gradation of the Cohen’s κ was <0.2 poor, 0.2–0.4 fair, 0.4–0.6 moderate 0.6–0.8 good, 0.8–1 very good (Landis and Koch, 1977). The Intra-class correlation coefficient (ICC value ranging from 0 to 1), defined as the proportion of variance of an observation due to between-subject variability in the true scores, was used to assess rating reliability by comparing the variability of different ratings of the same subject to the total variation across all ratings and all subjects. The type of ICC was 2,1 (Shrout and Fleiss, 1979). A high ICC indicates little variation between the scores assigned to each item by the raters. Gradation of ICC was 0–0.2 poor agreement, 0.3–0.4 fair agreement, 0.5–0.6 moderate agreement, 0.7–0.8 strong agreement, >0.8, almost perfect agreement (Shrout and Fleiss, 1979). Multivariate statistical analysis including PCA and OPLS-DA was carried out as described by Negri et al. (2017) for the LC-MS data and a *p*-value < 0.05 was considered statistically significant.

## Results

### Host Selection for Transient Expression and Optimization of the Infiltration Protocol

**FIGURE 1:**
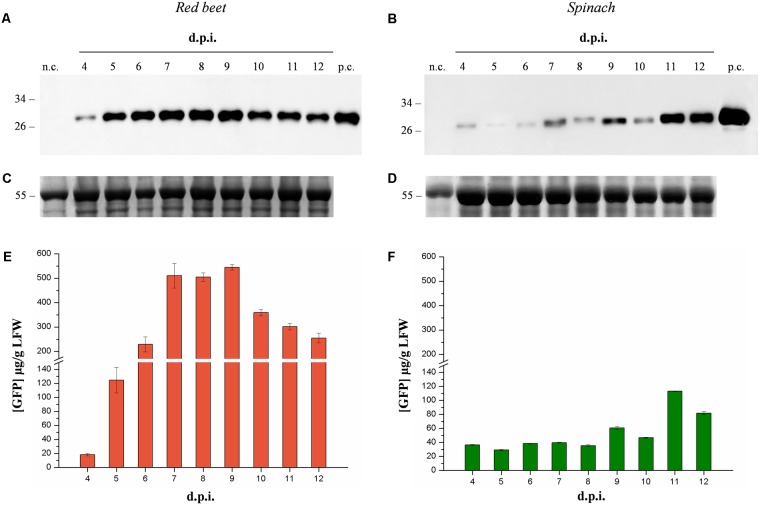
Time-course analysis of GFP expression in agroinfiltrated red beet and spinach leaves. Western blot analysis **(A,B)** and corresponding loading controls (RuBisCO large subunit) stained with Coomassie Brilliant Blue **(C,D)** of GFP-containing protein extracts from leaf samples collected from 4 to 14 dpi. The GFP content of each leaf protein extract was quantified by fluorescence measurement **(E,F)**. Left panels show samples from agroinfiltrated red-beet leaves, and right panels show samples from agroinfiltrated spinach leaves. Each lane was loaded with 3.5 and 30 μg of TSP for western blot and Coomassie staining, respectively. The western blot was probed with an anti-GFP antibody. Side numbers indicate molecular mass markers in kDa. p.c., positive control, 10 ng of commercial recombinant human GAD65; n.c., negative control, extract from leaves infiltrated solely with *A. tumefaciens* carrying magnICON^®^ 5′- and integrase modules.

We compared the suitability of the magnICON^®^ system for the production of recombinant proteins in the leaves of spinach (*Spinacia oleracea* cv Industra) and red beet (*Beta vulgaris* cv Moulin Rouge) plants initially using green fluorescent protein (GFP) as a model product. We conducted a time-course expression analysis and compared GFP levels on the day of maximum expression in each host. Suspensions of *A. tumefaciens* carrying GFP expression vectors were manually infiltrated into the leaves of both species and pools of three leaves were sampled daily, beginning 4 days post-inoculation (dpi) and finishing 12 dpi. Western blot analysis of leaf extracts and the quantification of GFP by fluorescence analysis revealed an expression peak 9 dpi in red beet leaves and 11 dpi in spinach leaves (**Figure 1**). The peak expression level of GFP in red beet was 544.9 ± 10.9 μg/g fresh leaf weight (FLW), which was significantly higher (Student’s *t*-test, *p* < 0.01) than in spinach (113.4 ± 0.3 μg/g FLW). We therefore selected red beet as the expression host for all subsequent experiments.

The infiltration protocol was switched to the vacuum method because this is more suitable for large-scale vaccine manufacturing (Chen et al., 2013). We tested two different vacuum infiltration parameters, namely the bacterial titer and the concentration of detergent.

**FIGURE 2:**
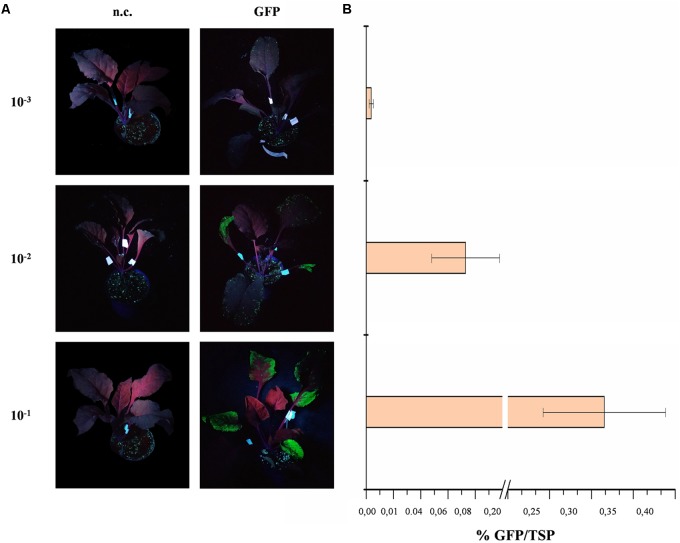
GFP expression in red beet following agroinfiltration with different *A. tumefaciens* titers. **(A)** UV illuminated images of infiltrated red beet leaves expressing GFP (right) and corresponding negative controls (n.c., left). **(B)** GFP content of infiltrated leaves quantified by fluorescence analysis of leaf protein extracts and shown as mean GFP percentage as a proportion of TSP in three biological replicates ± standard deviation.

We tested a dilution series of an overnight culture of *A. tumefaciens* ranging from 10^-1^ to 10^-3^ and found that higher bacterial titers produced greater GFP yields (**Figure 2**). The maximum GFP yield was achieved at the lowest dilution (10^-1^, equivalent to an OD_600_ of ∼0.35) and this was used in subsequent experiments. The concentration of Tween-20 in the infiltration buffer was tested in the range 0.005–0.05% because the amount of detergent has been shown to influence the protein yields in vacuum-infiltrated plants (Joh et al., 2005). However, UV illumination of the infiltrated plants and subsequent GFP quantification as above revealed no significant difference in protein yields (Student’s *t*-test, *p* < 0.01) among plants infiltrated with different detergent concentrations or in the absence of detergent (*data not shown*). However, the presence of the detergent reduced the amount of time required to infiltrate the plant tissue so we selected a mid-range concentration of 0.01% for further infiltration experiments.

### Selection of the Best-Performing Form of GAD and Immunological Characterization

Various forms of the target molecule GAD65 have previously been expressed in plants, and production has been optimized by expressing a catalytically inactive mutant form of the protein (GAD65mut) that retains the immunogenic properties necessary to induce oral tolerance (Avesani et al., 2010). More recently, an N-terminally truncated form of GAD65mut (Δ87GAD65mut) was expressed in *N. benthamiana* and tobacco (*N. tabacum*) to increase the solubility and stability of the protein. This form of the protein also accumulated at higher levels than its full-length counterpart when expressed in *N. benthamiana* using the magnICON^®^ system (Merlin et al., 2016).

**FIGURE 3:**
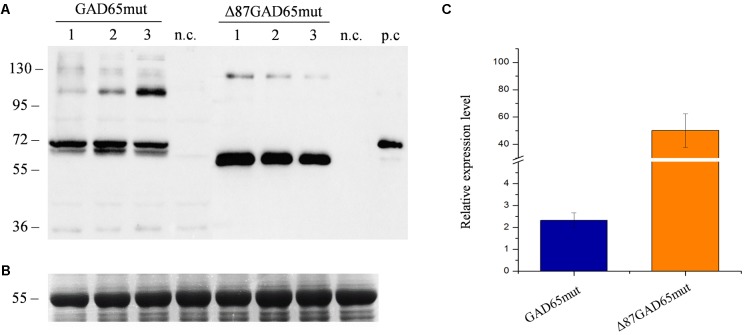
Comparison of GAD65mut and Δ87GAD65mut expression levels in red beet leaves. **(A)** Western blot analysis and **(B)** corresponding loading control (RuBisCO large subunit) stained with Coomassie Brilliant Blue, representing extracts from three red beet leaves expressing GAD65mut (left) and Δ87GAD65mut (right). The western blot was probed with an anti-GAD antibody (the lanes were loaded with 20 μL of extract for GAD65mut and 1 μL of extract for Δ87GAD65mut). Equal amounts of protein extract (10 μL/lane) were loaded for Coomassie staining. Side numbers indicate molecular mass markers in kDa. p.c., positive control, 10 ng of commercial recombinant human GAD65; n.c., negative control, extract from leaves solely infiltrated with *A. tumefaciens* carrying magnICON^®^ 5′ and integrase modules. **(C)** Relative expression levels of the two protein forms, plotted as mean values of densitometric analysis of western blot image, using positive control band as reference.

The same magnICON^®^ vectors used in our previous study (Merlin et al., 2016) were used here to express GAD65mut and Δ87GA65mut in red beet leaves. A time-course analysis from day 2 to 14 revealed that both protein forms were expressed but with different yields and accumulation profiles (Supplementary Figure 1). The peak expression of GAD65mut was 5 dpi and that of Δ87GAD65mut was 11 dpi (**Figure 3**). Furthermore, the peak expression level of Δ87GAD65mut was more than 20-fold higher than its full-length counterpart (**Figure 3**). The truncated protein was therefore selected as the preferred T1D oral vaccine candidate.

**FIGURE 4:**
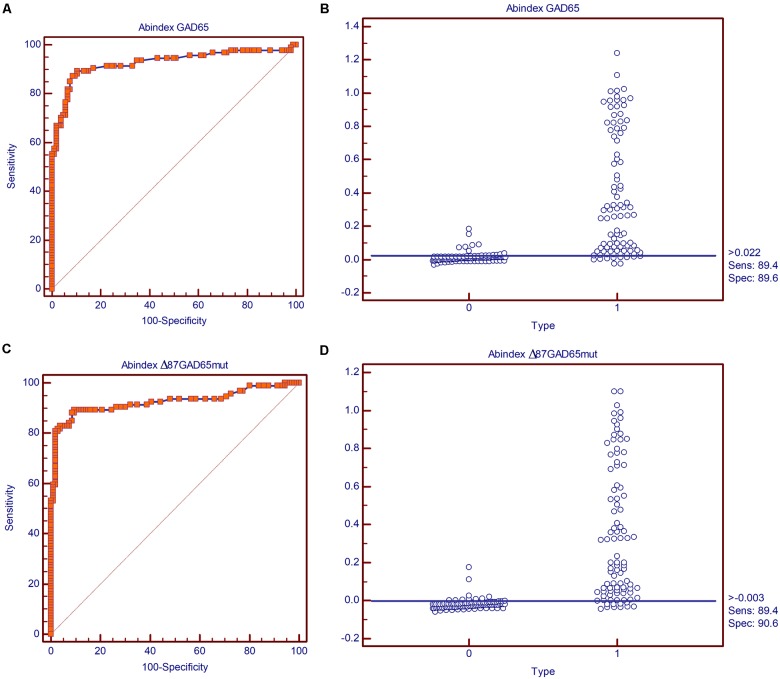
Diagnostic sensitivity and specificity of the GAD65Ab and Δ87GAD65mut assays. Receiving operator characteristic (ROC) curves for the **(A)** GAD65Ab and **(C)** Δ87GAD65mutAb assays, and classification of negative and positive samples by the best cut-off value according to the Youden index for the **(B)** GAD65Ab and **(D)** Δ87GAD65mutAb assays.

The immunoreactivity of Δ87GAD65mut was compared to that of the full-length wild-type protein (GAD65) by radioimmunoassay using sera from 94 new-onset T1D patients and from 106 healthy control subjects. This analysis is based on protein recognition by autoantibodies present in sera from T1D patients, thus providing insight into the immunological features and conformation of the proteins. The immunoreactivity of the two molecules was evaluated in terms of their diagnostic sensitivity and specificity by receiver operating characteristic (ROC) curve analysis to distinguish T1D and control samples. Using the best cut-off value calculated according to the Youden index (0.022 for the GAD65 antibody assay and -0.003 for the Δ87GAD65mut antibody assay) both assays achieved satisfactory diagnostic accuracy, with area under the curve (AUC) values and corresponding 95% confidence intervals (95% CI) of 0.927 for GAD65 (95% CI 0.882–0.959) and 0.924 for Δ87GAD65mut (95% CI 0.879–0.957) and no statistically significant differences (*p* = 0.8696) between them (**Figure 4**).

The concordance of qualitative results (classification of subjects as positive or negative by the two assays) showed agreement in 85 of 94 (90%) T1D sera and 104 of 106 (98%) healthy control sera, with a very good Cohen’s κ coefficient of 0.874 (95% CI 0.802–0.946) for the general agreement. To determine the inter-assay concordance for GAD65 antibody levels, the intraclass correlation coefficient (ICC, ranging from 0 to 1) was calculated for all assays couples. In this type of analysis, a high ICC indicates that there is little variation among GAD65 antibody levels in each serum sample as determined by the two assays, and we observed strong to almost perfect agreement (ICC = 0.951).

### Oral Vaccine Candidate Design

The combination of Δ87GAD65mut as the preferred product, red beet as the preferred host and the corresponding best-performing protocol for transient expression in terms of recombinant protein accumulation was selected for the production of a T1D oral vaccine candidate. The concentration of Δ87GAD65mut was measured by densitometry on western blots of red beet leaf extracts using known amounts of purified recombinant GAD to produce a standard curve. The truncated protein form accumulated to levels of 201.4 ± 29.3 μg/g FLW in red beet leaves at 11 dpi (Supplementary Figure 2).

**FIGURE 5:**
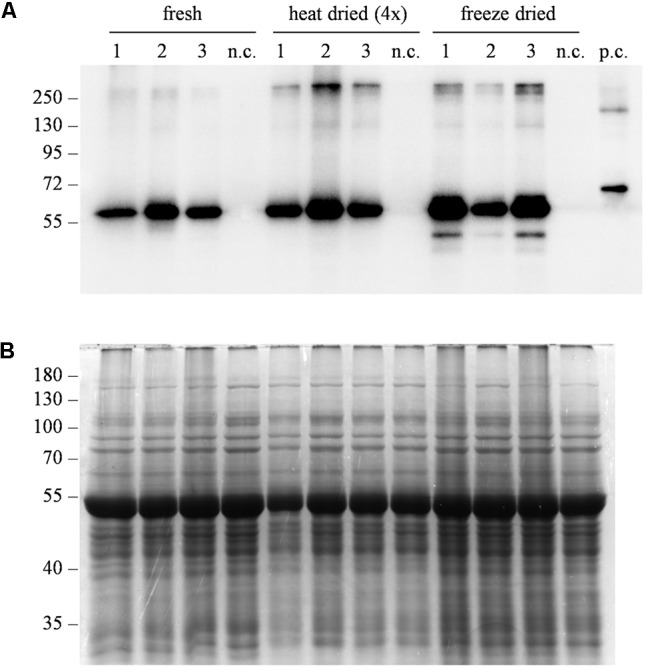
Evaluation of protein stability after different dehydration treatments. **(A)** Western blot analysis and **(B)** corresponding gel stained with Coomassie Brilliant Blue representing three independent extracts from leaves expressing Δ87GAD65mut. After sampling, leaves were directly frozen (fresh), heat-dried at 50°C for 10–11 h (heat dried) or lyophilized at –50°C, 0.04 mBar for 72 h (freeze dried). Total soluble proteins were extracted at different tissue:buffer ratios to consider water loss due to dehydration. Different amounts of protein extracts were loaded for western blot analysis: 0.25 μL/lane for the “fresh” and “freeze-dried” extracts, 1 μL of “heat-dried” extract. Equal amounts of protein extract (10 μL/lane) were loaded for Coomassie staining. The western blot was probed using an anti-GAD antibody. Side numbers indicate molecular mass markers in kDa. n.c., negative control, extract from leaves infiltrated solely with the *A. tumefaciens* carrying magnICON^®^ 5′ and integrase modules.

One of the challenges associated with plant-derived oral vaccines is to ensure that the integrity of the immunoreactive target molecule is maintained in the plant tissue used for oral administration. Plant-derived biopharmaceuticals for oral delivery are often administered as minimally processed dried biomass (Merlin et al., 2017). To evaluate the stability of Δ87G AD65mut in dehydrated leaves, western blot analysis was carried out after freeze-drying/lyophilization or heat-drying treatments, in comparison to untreated (only frozen) tissue (**Figure 5**). The dehydration of fresh leaf material reduced the tissue weight by ∼90% due to the removal of water from the plant cells (*data not shown*) and maintained the integrity of target protein. However, freeze-drying/lyophilization achieved a higher yield of immunoreactive protein than heat drying (**Figure 5**).

Another important challenge associated with oral vaccines is to ensure the delivery of bioactive molecules to the gut-associated lymphoid tissue (GALT), which means that the structural integrity of the vaccine must be maintained as it moves through the gastric tract. Plant cells are thought to facilitate this by encapsulating recombinant antigens and protecting them from degradation caused by the low pH and digestive enzymes in the stomach (Kohli et al., 2014; Su et al., 2015; Xiao et al., 2016). We therefore tested the stability of Δ87GAD65mut in plant tissue after exposure to low pH treatment and a simulation of gastric digestion.

We hypothesized that plant cells remain intact after tissue grinding and dehydration, and therefore the intracellular proteins should not be released in the extraction buffer and hence would be protected from acidic conditions that would otherwise cause them to precipitate (Fink et al., 1994). Western blot analysis of the soluble and insoluble fractions produced when dried plant tissue was resuspended in buffer with a near neutral pH revealed that Δ87GAD65mut was partially solubilized, suggesting that leaf grinding and drying disrupts at least some cells (Supplementary Figure 3A). Under acidic conditions, the Δ87GAD65mut signal was observed only in the insoluble fraction, probably reflecting the protein content of unbroken cells, whereas the solubilized Δ87GAD65mut precipitated due to low pH (Supplementary Figure 3A). The distribution of Δ87GAD65mut in the different samples matched the distribution of total soluble protein, as demonstrated by staining the gel with Coomassie Brilliant Blue (Supplementary Figure 3B). These results confirmed that freeze-drying/lyophilization was the best treatment for the preparation of the vaccine candidate.

**FIGURE 6:**
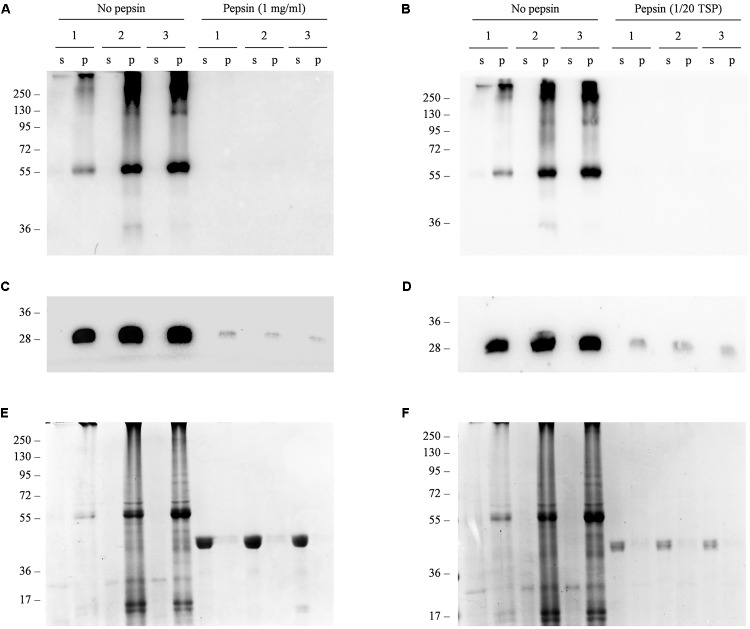
*In vitro* simulated gastric digestion of Δ87GAD65mut in freeze-dried red beet leaves. **(A–D)** Western blot analysis and **(E,F)** corresponding gels stained with Coomassie Brilliant Blue representing three independent extracts of leaves expressing Δ87GAD65mut after simulated gastric digestion. Pepsin was added at a final concentration of 1 mg/mL **(A,C,E)** or at a ratio of 1:20 to the TSP **(B,D,F)** to 100 mg of lyophilized tissue. Samples without enzyme were used as controls (no pepsin). Supernatants (s) and pellets (p) obtained in the final centrifugation after treatment were loaded for SDS-PAGE analysis (24 and 16 μl, respectively). The western blot was probed with anti-GAD **(A,B)** and anti-LHCB2 **(C,D)** antibodies. Side numbers indicate molecular mass markers in kDa.

Next, we carried out a simulated acidic digestion of the freeze-dried material using two different concentrations of the porcine gastric enzyme pepsin, based on previously published reports (Elless et al., 2000; Schulz et al., 2015). Western blot analysis of the digested samples demonstrated that the recombinant protein was degraded under both treatment conditions (**Figure 6**). We carried out an additional western blot to detect the endogenous antenna complex protein LHCB2, which is localized within the chloroplast thylakoids and is thus protected by multiple subcellular membranes. We did not detect a signal for this protein either, indicating that even subcellular compartments lose their integrity under acid digestion conditions and that targeting Δ87GAD65mut to accumulate within a subcellular compartment is unlikely to protect it from degradation.

We also tested the agroinfiltrated red-beet leaves for residual bacterial load by plating resuspended freeze-dried tissue onto growth medium in the presence of different antibiotics. Our results indicated that the treatment used to prepare the oral vaccine candidate completely removed any bacterial bioburden from the samples (**Table 1**).

**Table 1:** Residual bacterial charge analysis.

	Residual bacterial charge				
	No antibiotics	Rifampicin	Rifampicin Carbenicillin	Rifampicin Kanamycin	Rifampicin Carbenicillin Kanamycin
Δ87GAD65mut	0	0	0	0	0
Wild-type	0	0	0	0	0

*CFU/mL recorded when extracts of 100 mg freeze-dried red beet leaves expressing Δ87GAD65mut and non-infiltrated controls were incubated for 3 days at 28°C on LB medium without antibiotics and containing (i) 50 μg/mL rifampicin, (ii) 50 μg/mL each of rifampicin and carbenicillin, (iii) 50 μg/mL each of rifampicin and kanamycin, or (iv) 50 μg/ml each of rifampicin, carbenicillin and kanamycin. The analysis was performed using three biological replicates for each sample.*

### Assessment of the Metabolic Bioequivalence of Δ87GAD65mut and Control Red Beet Plants

Fingerprints of primary and secondary metabolites and polar lipids were generated by liquid chromatography mass spectrometry (LC-MS) from nine red beet plants transiently expressing Δ87GAD65mut, nine agroinfiltrated controls (infiltrated with wild-type *A. tumefaciens*) and nine wild-type controls (no infiltration).

**FIGURE 7:**
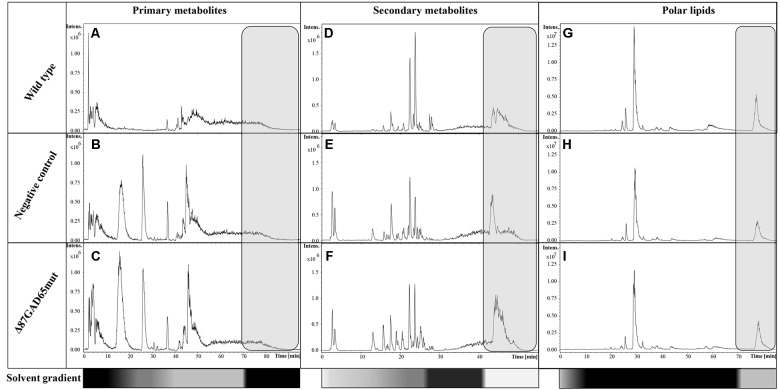
LC-MS base peak chromatograms of wild-type, negative control and Δ87GAD65mut-expressing plants. The data were acquired in negative ionization mode. Primary metabolites **(A–C)**, secondary metabolites **(D–F)**, and the lipids **(G–I)** are shown for one wild-type, negative control and Δ87GAD65mut plant each. The gray boxes show the chromatographic zone that was not excluded from analysis. The bottom bars highlight the solvent gradients in terms of B solvent percentage (white: 0%; gray: 50%; black: 100%).

**FIGURE 8:**
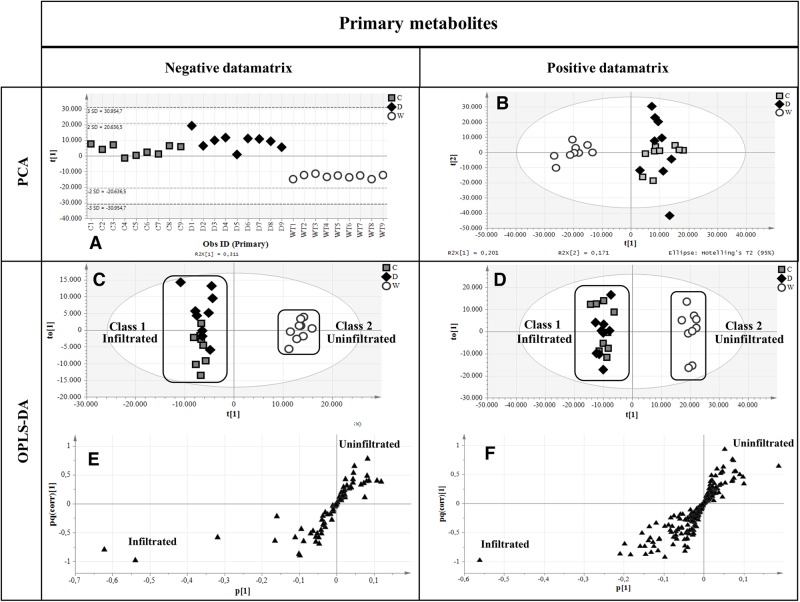
Multivariate statistical analysis outputs for primary metabolites. **(A,B)** PCA score scatter plots of primary metabolites analyzed in negative and positive ionization mode. **(C,D)** OPLS-DA score plots highlighting the two imposed classes. White circles, wild-type plants; gray boxes, negative controls; black diamonds, Δ87GAD65mut-expressing plants. **(E,F)** S-loading plots showing metabolites correlating [pq(corr)] with a specific class. Black triangles, the putative metabolites.

**FIGURE 9:**
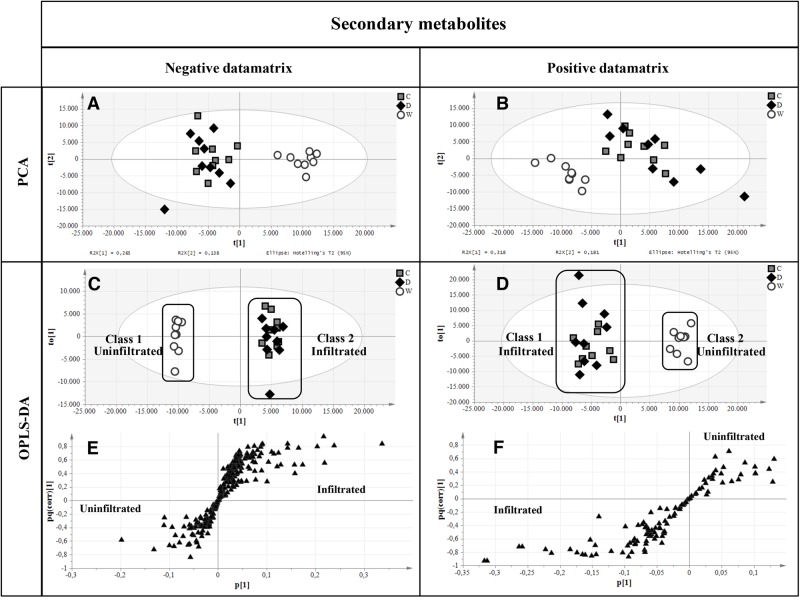
Multivariate statistical analysis of secondary metabolites analyzed in negative and positive ionization modes. **(A,B)** PCA score plots. **(C,D)** OPLS-DA score plots with the two attributed classes. **(E,F)** S-loading plots showed the most correlating putative metabolites. Symbols are the same as explained in **Figure 8**.

Primary metabolites were analyzed by hydrophilic interaction liquid chromatography (HILIC)-MS, whereas secondary metabolites and polar lipids were analyzed by reversed-phase (RP)LC-MS. The chromatograms are shown in **Figure 7** and Supplementary Figure 4. The metabolic profiles were then processed by unsupervised principal component analysis (PCA). For the primary metabolites, one principal component explained 31.1% of the total variance for the negative dataset (**Figure 8A**) whereas three principal components explained 47.6% of the total variance for the positive dataset (**Figure 8B**). For the secondary metabolites, two principal components explained 40.3% of the total variance in the negative dataset (**Figure 9A**), whereas three principal components explained 60.7% of the total variance in the positive dataset (**Figure 9B**). For both the primary and secondary metabolites, PCA revealed no separation between the Δ87GAD65mut and infiltrated negative control plants, but the wild-type plants formed a separate cluster. Orthogonal projections to latent structures discriminant analysis (OPLS-DA) was then used to compare the infiltrated and non-infiltrated plants in terms of both primary (**Figures 8C,D**) and secondary (**Figures 9C,D**) metabolites. The S-loading plots revealed primary metabolites (**Figures 8E,F** and **Table 2**) and secondary metabolites (**Figures 9E,F** and **Table 2**) with pq(corr) values lower than -0.8 or higher than 0.8, indicating specific classes (Zampieri et al., 2014). The imposition of three different classes (Δ87GAD65mut vs. agroinfiltrated control vs. wild-type) or two classes (Δ87GAD65mut vs. agroinfiltrated control, excluding wild-type) showed no statistically significant differences among the groups (*data not shown*). Finally, PCA revealed no clustering within the positive or negative data matrices representing the polar lipid extracts (Supplementary Figure 5).

**Table 2:** Putative class-characterizing metabolites.

ID	m/z	rt	pq(corr)
**Primary metabolites**			
**Negative datamatrix**			
4	193,76	26,03	-0,979665
46	388,72	25,95	-0,894626
44	367,59	26,64	-0,872155
**Positive datamatrix**			
2	195,82	25,97	-0,982993
90	391,09	26,02	-0,924073
11	210,04	45,21	-0,886428
36	193,00	45,17	-0,873365
25	254,12	26,34	-0,868975
47	333,12	26,61	-0,841397
208	218,23	25,88	-0,821996
80	254,13	27,43	-0,815249
220	159,06	44,42	0,933643
**Secondary metabolites**			
**Negative datamatrix**			
249	212,90	27,98	-0,839398
104	386,66	13,55	0,807645
4	190,71	3,27	0,813981
67	410,91	2,64	0,82496
15	110,81	3,35	0,835718
6	536,03	2,60	0,836376
85	416,88	24,30	0,836501
11	193,71	2,79	0,945718
**Positive datamatrix**			
9	343,07	2,67	-0,93146
7	196,08	2,75	-0,928641
76	493,21	21,97	-0,868173
43	560,18	2,71	-0,855664
28	329,35	2,77	-0,841778
26	218,31	2,59	-0,830092
31	423,17	16,75	-0,829772
22	348,93	3,16	-0,813145
78	428,84	2,69	-0,808734
29	447,17	24,56	-0,803155

*Legend: m/z: mass to charge ratio; rt: retention time.*

*Comparison of putative primary and secondary metabolites characterizing a specific class [pq(corr) < -0.8 and >+0.8] between infiltrated and non-infiltrated (wild-type) plants.*

## Discussion

More than 100 clinical trials for the treatment of autoimmune diabetes are currently registered on www.clinicaltrials.gov, including more than 30 using immunoregulatory strategies to stop or prevent the destruction of pancreatic beta cells. One of these trials involved the oral administration of insulin for the prevention of T1D in relatives at risk of developing the disease (NCT00419562). The amount of oral insulin administered in this study is 7.5 mg per day for 7–8 years or until the required amount of information has been gathered. The first results indicate an average 31 months delay in the progression to T1D in a subset of 55 subjects among 560 enrolled individuals (Greenbaum, 2017). This clinical program was possible thanks to the cost-effective production of large amounts of insulin in recombinant microbes (Baeshen et al., 2014) and the dramatic results obtained in a subset of patients paves the way toward future immunomodulatory strategies using other T1D autoantigens such as GAD65. The oral administration of GAD65 is likely to be particularly beneficial in subjects that test positive for autoantibodies against GAD65 but not insulin, because such individuals are not expected to respond to oral insulin. Furthermore, combinatorial approaches involving the concomitant administration of multiple autoantigens would extend the protective benefits of oral tolerance and would address subjects testing positive for more than one type of autoantibody (Lernmark and Larsson, 2013). However, massive quantities of clinical-grade autoantigens would be required to test this approach (several kilograms of pure protein for each phase III clinical trial), so a cost-effective and highly efficient production platform for recombinant autoantigens would be beneficial for clinical development and would be mandatory should the approach be rolled out more generally.

Insulin is a relatively simple polypeptide and microbial production is adequate for this product. In contrast, GAD65 is a more complex protein and alternative production platforms are required for the cost-effective manufacture of sufficient quantities of GAD65 with the necessary quality attributes (Merlin et al., 2014). Plants provide a versatile platform for the production of recombinant proteins, particularly for oral vaccine development, because edible plant tissues can be used as a vehicle for vaccine administration without extensive processing and purification. Many edible species and plant organs have been considered as platforms for recombinant protein production, and the ability to express proteins in the leaves of some edible species using the deconstructed magnICON^®^ expression system based on Tobacco mosaic virus has already been demonstrated (Marillonnet et al., 2005).

We previously used the magnICON^®^ system to demonstrate proof of principle for the expression of human GAD65 derivatives in *N. benthamiana* (Merlin et al., 2016). However, *N. benthamiana* is not generally regarded as an edible species due to the alkaloids and other metabolites that accumulate in the leaves, and alternative crops are needed for the routine production of oral vaccines. We therefore compared two edible crops – spinach and red beet – for their ability to produce recombinant proteins efficiently, using GFP as a model. Red beet was the most suitable, achieving higher yields of GFP with our standard agroinfiltration method, perhaps reflecting the larger intercellular spaces in beet leaves compared to spinach (*data not shown*). We then tested a range of vacuum agroinfiltration parameters to determine the optimal protocol in red beet, given that the standard protocol is optimized for tobacco species, which have even larger intercellular spaces than red beet (Marillonnet et al., 2005). We found that optimal infiltration was achieved with a higher bacterial titer than typically used in tobacco, but that the yields were not as high. The concentration of detergent did not appear to play a significant role in the efficiency of expression and we therefore chose conditions that allowed infiltration to be completed in the shortest time.

The form of GAD for expression was selected by comparing GAD65mut with the truncated form Δ87GAD65mut, the latter providing to be superior in red beet as previously reported in *N. benthamiana* plants agroinfiltrated with magnICON^®^ vectors (Merlin et al., 2016). We have previously confirmed the immunoreactivity of Δ87GAD65mut but a comparison with the full-length wild-type counterpart has not been reported. This is an important control reference because wild-type GAD65 is currently being used in human clinical trials and for T1D diagnosis. Accordingly, we found that wild-type GAD65 and Δ87GAD65mut behave in a similar manner in terms of immunoreactivity toward conformational autoantibodies present in T1D patient sera, indicating a similar conformation and folding of the two proteins. These data confirmed a similar immunoreactivity of the two proteins indicating that Δ87GAD65mut is suitable as an oral vaccine candidate.

The average Δ87GAD65mut expression level in red beet leaves was 201.4 ± 29.3 μg/g FLW, which is sufficient to commence the development of a T1D oral vaccine that will be required in large amounts, ranging from micrograms per day per animal for preclinical studies in mouse models of T1D through to milligrams per day per subject for human clinical trials, as previously reported for recombinant oral insulin (Polanski et al., 1997; Skyler et al., 2005). The average recombinant protein levels produced in red beet leaves are consistent with previous results in edible plant systems (Merlin et al., 2017). However, our novel platform has several advantages. First, the magnICON^®^ system produces the recombinant protein in a much shorter timeframe (up to 2 weeks) compared to transgenic or transplastomic lettuce, rice, carrots and maize (Uvarova et al., 2013; Fukuda et al., 2015; Nahampun et al., 2015; Su et al., 2015). Second, the magnICON^®^ system achieves higher yields than other transient expression systems in edible species (Clarke et al., 2017). The production of a massive amount of leaf tissue expressing the recombinant protein for oral tolerance induction would therefore require commensurate facilities for plant cultivation. Furthermore, the magnICON^®^ system requires the continuous infiltration of plant material. However, the protocol described herein is compatible with continuous production and easily scalable, making it ideal for the production of a T1D oral vaccine. On the basis of the yields we achieved and the 70,000 children diagnosed each year with T1D (International Diabetes Federation, 2006), we estimate that 42,000 red beet plants (corresponding to 200 m^2^ of greenhouse space) would be sufficient to meet global demand for a T1D secondary prevention strategy, assuming treatment comprises the daily administration of 1 mg of Δ87GAD65mut for 8 years.

Leafy tissues comprise more than 95% water, and this environment encourages protein degradation and also the proliferation of microbes. The effective delivery of large doses of recombinant protein would therefore ideally be achieved using dried plant tissue, to facilitate volume reduction, to increase shelf life, and to reduce the bioburden. Our results indicate that lyophilization meets all these requirements by reducing the leaf water content by at least 10-fold (*data not shown*), by preserving the high Δ87GAD65mut yields recorded before treatment, and by eliminating bacteria, including the recombinant *A. tumefaciens* used during production. Although contamination by viable microbes (bioburden) can be controlled, the possibility exists that the processed leaf tissue may carry bacterial lipopolysaccharide (LPS; endotoxin). The highest source of potential residual endotoxin in the process described would be from the vector, as *Agrobacterium* is a Gram-negative bacterium with LPS present in its cell wall. It is important to emphasize that although LPS is highly bioactive systemically in humans and other mammals, it is not considered a high safety risk if administered orally. Harper et al. (2011) showed that up to 1 million endotoxin units (EU) of LPS can be administered safely to mice by oral gavage with no evidence of toxicity as assessed by body weight effects, clinical signs, gross organ lesions at necropsy, etc. Similarly, Wallace et al. (2016) reviewed the safety of endotoxins on feed and occupational exposure in people and found the risk to be low if exposure is via the oral route. This is not surprising since humans are constantly exposed to endotoxin from the 10^12^ bacterial cells/ml in their colon and have likely become tolerized to LPS via commensalism (d’Hennezel et al., 2017).

In this proof-of-principle study, we used a relatively high ratio of inoculum to plant tissue and induced gene expression via agroinfiltration. In an industrial setting, the production of large quantities of GAD65 to meet market demand could be achieved in transgenic red beet plants carrying the GAD65 gene under control of an ethanol-inducible promoter (Werner et al., 2011). Although transgenic lines remain to be constructed, such a system offers the twin advantages of obviating (a) the cost of manufacturing the agrobacterial inoculum by replacing it with a low-cost inducer (e.g., dilute ethanol), and (b) the vector-borne endotoxin.

The treated leaf tissue was evaluated to determine whether the Δ87GAD65mut protein was protected by bioencapsulation, but our results indicated that the protein content of the tissue was digested under the conditions found in the gastric environment. We hypothesized that the lyophilization treatment damages the plant cell wall and exposes the Δ87GAD65mut protein to the acidic milieu and digestive enzymes present in the stomach. Previous studies have shown that the oral delivery of powdered tobacco leaves (not lyophilized) expressing a cholera toxin fusion protein in the chloroplast makes the recombinant protein bioavailable in the liver, intestinal mucosa and spleen, but quantitative bioavailability data were not presented (Limaye et al., 2006).

Our results suggest that, for the proposed application, the freeze-dried/lyophilized leaf material should be formulated in order to overcome the gastric environment and to be released in the intestine. Recent studies with oral delivered insulin demonstrated that synthetic hydrogels, three-dimensional mesh like networks containing hydrophilic polymers (Renukuntla et al., 2013), may fulfill these requirements (Tuesca et al., 2008).

PCA was used to evaluate the metabolic bioequivalence of plants expressing Δ87GAD65mut compared to infiltrated and non-infiltrated controls, revealing no significant difference between the infiltrated plants expressing Δ87GAD65mut and the infiltrated controls, whereas the non-infiltrated wild-type plants formed a separate cluster. This shows that certain primary and secondary metabolites distinguish infiltrated plants from untreated plants, but the presence of Δ87GAD65mut has little overall impact. These results may indicate that the LC-MS analysis identified bacterial metabolites, or metabolites produced by the infiltrated plants as defense/stress-response signals. The infiltration of red beet plants with *A. tumefaciens* (Hellens et al., 2000) triggers the production of reactive oxygen species (ROS) after 24 h (Sepúlveda-Jiménez et al., 2005). In turn, ROS are known to induce defense mechanisms that modulate the levels of certain metabolites. Among the secondary metabolites identified by PCA, sinapic acid and kaempferol derivatives protect plants against UV-B stress (Li et al., 1993), and anthocyanins acylated with caffeic acid and certain caffeic and coumaric acid derivatives might protect cultured plant cells from heat stress (Commisso et al., 2016). There was no significant difference among the three groups of plants in terms of polar lipid profiles, indicating that such metabolites are not affected by agroinfiltration or the expression of Δ87GAD65mut.

Overall, our results suggest that freeze-dried/lyophilized red beet plants are suitable for the safe and cost-effective production of vaccine candidates for the future clinical testing of T1D oral immunotherapy.

## Author Contributions

EB performed the transient expression in red beet plants and their biochemical characterization. MM contributed in manuscript writing and supervised the biochemical characterization of red beet plants. EG supervised the upstream process set-up and the statistical analysis. AP performed the transient expression in spinach plants. AB performed the radiobinding assay. MC performed the bioequivalence study and wrote the corresponding sections. AF supervised all the immunological experiments and critically revised the manuscript. VB performed the statistical analysis of the radiobinding assay. VK supervised the expression in the MagnICON system. MP contributed in manuscript writing and in its critical revision. LA designed all the experiments, coordinated all the experimental activities, and wrote the manuscript. She is the principal investigator of the project that was funded and supported all the experimental activities.

## Conflict of Interest Statement

The authors declare that the research was conducted in the absence of any commercial or financial relationships that could be construed as a potential conflict of interest.

## Footnotes

1 http://mzmine.github.io/
