# Supplementary material for: Design of a Type-1 Diabetes Vaccine Candidate Using Edible Plants Expressing a Major Autoantigen
Source: Front Plant Sci. 2018 May 1;9:572. doi: 10.3389/fpls.2018.00572 (PMC5938395; doi:10.3389/fpls.2018.00572)
Supplement: Supplementary file 1 [file Table_1.DOCX]

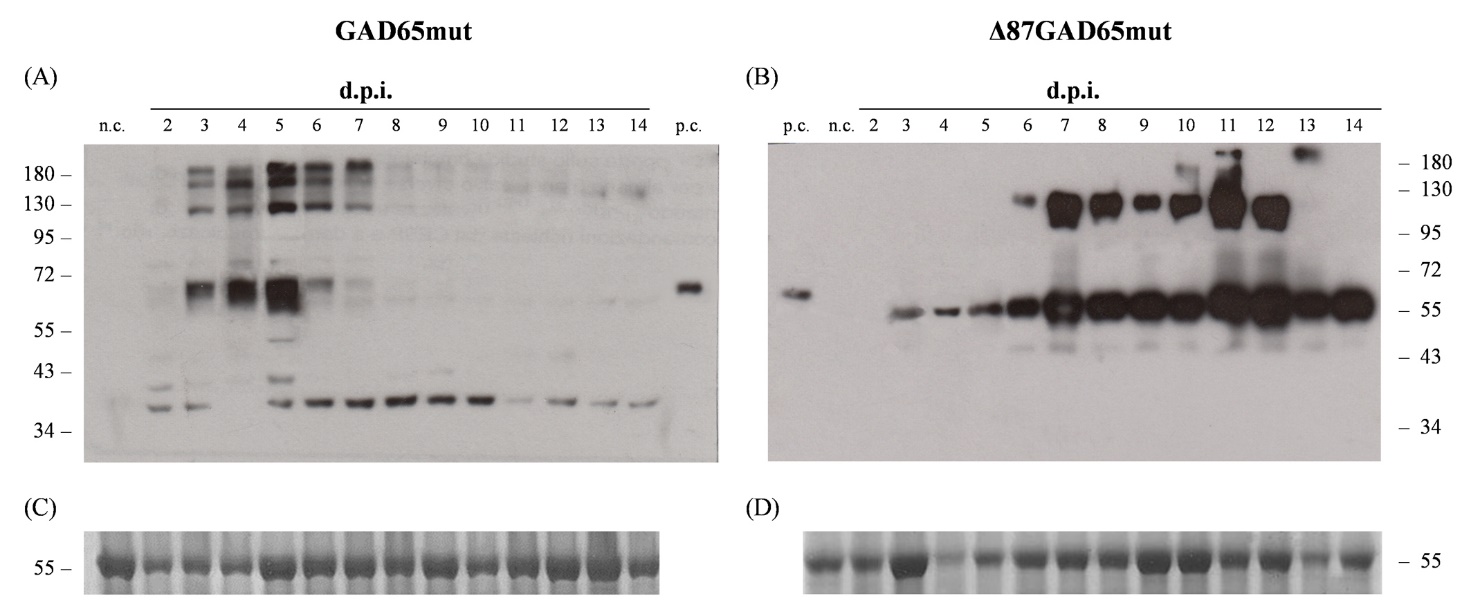


**Supplementary Figure 1:** Time-course analysis of GAD expression in agroinfiltrated red beet leaves. **(A,B)** Western blot analysis and **(C,D)** corresponding loading control (RuBisCO large subunit) stained with Coomassie Brilliant Blue, representing protein extracts of leaves expressing GAD65mut (right panels) and Δ87GAD65mut (right panels) collected from 2 to 14 dpi. The western blot was probed with an anti-GAD antibody (the lanes were loaded with 20 μL of extract for GAD65mut and 1 μL of extract for Δ87GAD65mut). Equal amounts of protein extract (10 μL/lane) were loaded for Coomassie staining. Side numbers indicate molecular mass markers in kDa. Abbreviations: p.c. = positive control, 10 ng of commercial recombinant human GAD65; n.c. = negative control, extract from leaves infiltrated solely with the *A. tumefaciens* carrying magnICON® 5′ and integrase modules.


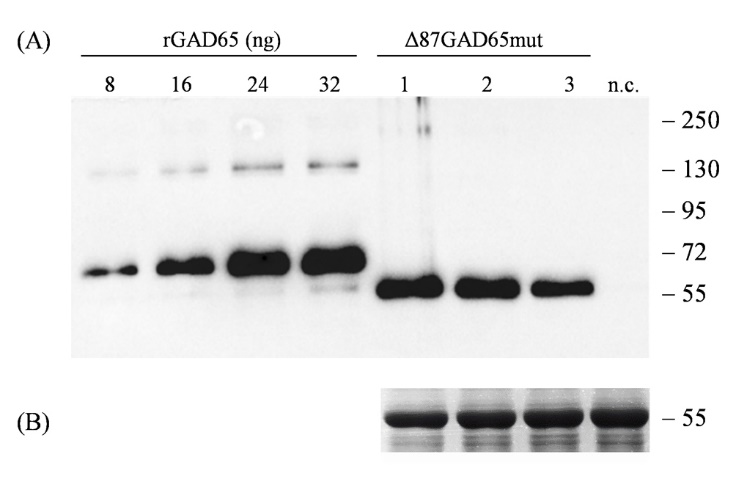


**Supplementary Figure 2**: Quantification of Δ87GAD65mut expressed in red beet leaves. **(A)** Western blot analysis and **(B)** corresponding loading control (RuBisCO large subunit) stained with Coomassie Brilliant Blue, representing three independent extracts of leaves expressing Δ87GAD65mut compared to different amounts of commercial recombinant human GAD65. The western blot was probed with an anti-GAD antibody (the lanes were loaded with 0.5 μL/lane of Δ87GAD65mut extracts and 8, 16, 24 or 32 ng of commercial recombinant human GAD). Equal amounts of protein extract (10 μL/lane) were loaded for Coomassie staining. Side numbers indicate molecular mass markers in kDa. Abbreviations: n.c. = negative control, extract from leaves infiltrated solely with the *A. tumefaciens* carrying magnICON® 5′ and integrase modules.


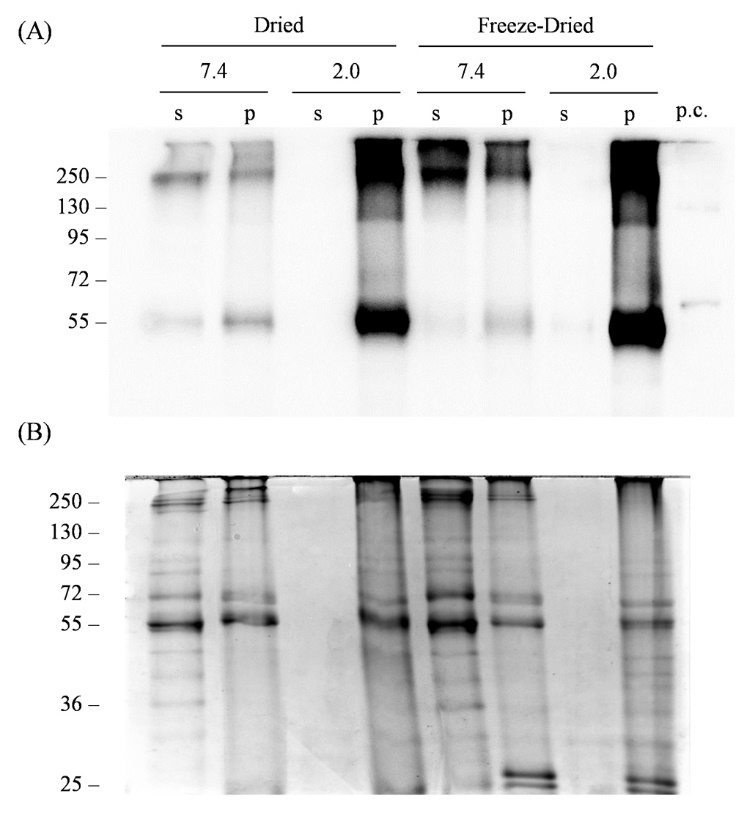


**Supplementary Figure 3**: Evaluation of the effects of low pH on soluble and insoluble protein fractions. **(A)** Western blot analysis and **(B)** corresponding gel stained with Coomassie Brilliant Blue, representing soluble (s) and insoluble (pellet, p) fractions from freeze-dried and heat-dried extracts of leaves expressing Δ87GAD65mut obtained under different pH conditions. Equal amounts of extract were loaded for western blot analysis and gel staining(10 μL/lane). The western blot was probed with an anti-GAD antibody. Side numbers indicate molecular mass markers in kDa. Abbreviations: n.c. = negative control, extract from leaves infiltrated solely with the *A. tumefaciens* carrying magnICON® 5′ and integrase modules.


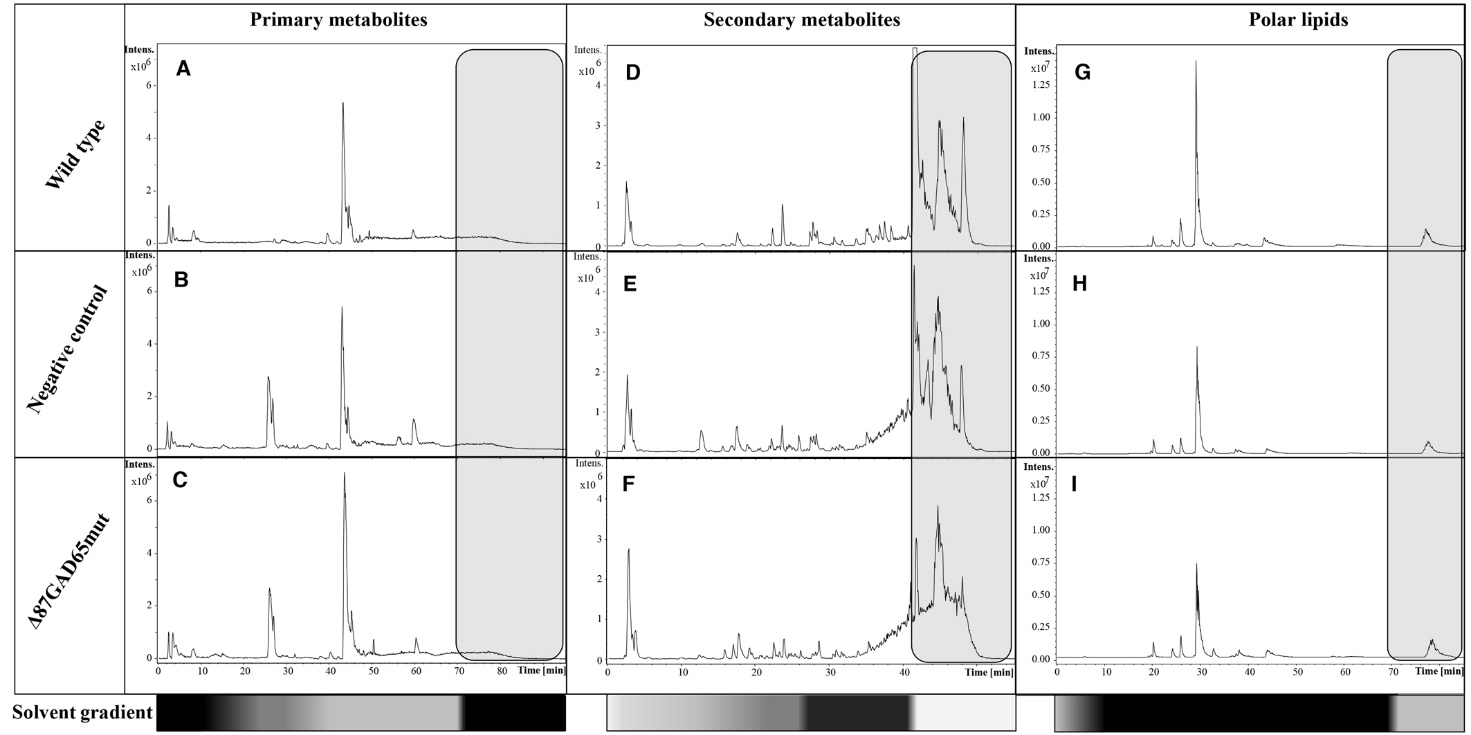


**Supplementary Figure 4**: Positive LC-MS base peak chromatograms. Rows indicate the sample type (wild type, negative control and plants expressing Δ87GAD65mut). **(A-C)** Primary metabolites, **(D-F)** secondary metabolites, **(G-I)** lipids. Peaks included in the gray boxes were excluded from analysis. The solvent gradient bars show the percentage of solvent B in grayscale, from white (0%) to black (100%).


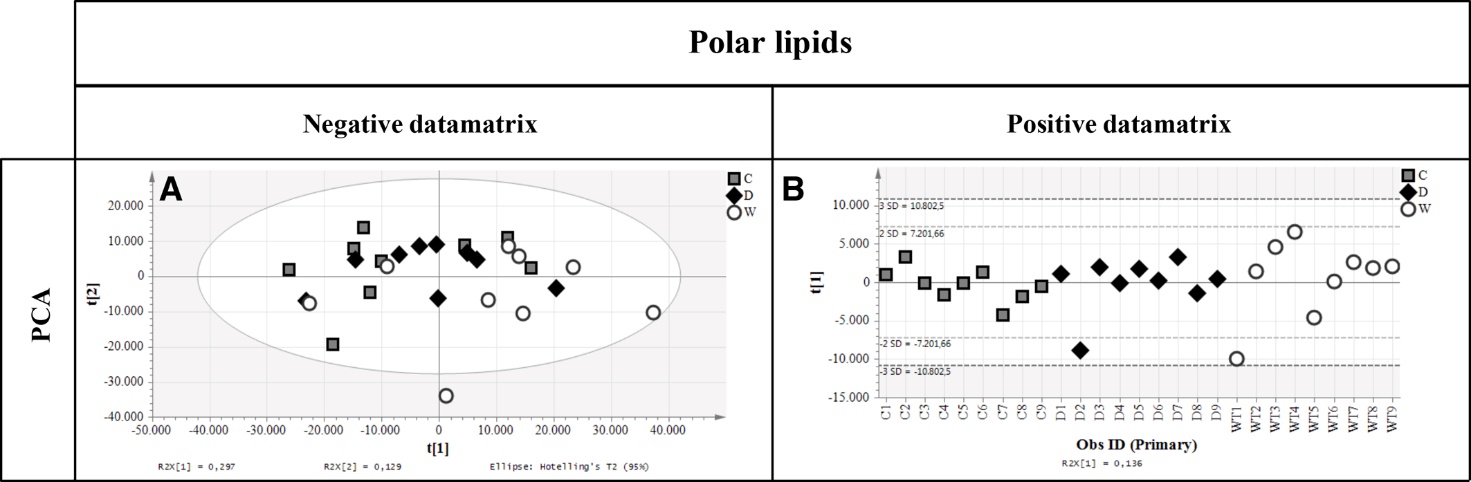


**Supplementary Figure 5**: PCA score scatter plots for polar lipids. The negative **(A)** and positive **(B)** LC-MS panels show the distributions for lipids in wild-type (white circles), negative control (gray boxes) and Δ87GAD65mut-expressing (black diamonds) plants.
